# Influence of SiO_2_ Nanoparticles Extracted from Biomass on the Properties of Electrodeposited Ni Matrix Composite Films on Si(100) Substrate

**DOI:** 10.3390/ma17164138

**Published:** 2024-08-21

**Authors:** Ivana O. Mladenović, Nebojša D. Nikolić, Vladislav Jovanov, Željko M. Radovanović, Marko M. Obradov, Dana G. Vasiljević-Radović, Marija M. Vuksanović

**Affiliations:** 1 Institute of Chemistry, Technology and Metallurgy, University of Belgrade, Njegoševa 12, 11 000 Belgrade, Serbia; nnikolic@ihtm.bg.ac.rs (N.D.N.); vladislav.jovanov@ihtm.bg.ac.rs (V.J.); marko.obradov@ihtm.bg.ac.rs (M.M.O.); dana@nanosys.ihtm.bg.ac.rs (D.G.V.-R.); 2 Innovation Centre of Faculty of Technology and Metallurgy doo, Karnegijeva 4, 11 000 Belgrade, Serbia; zradovanovic@tmf.bg.ac.rs; 3 “VINČA” Institute of Nuclear Sciences—National Institute of the Republic of Serbia, Department of Chemical Dynamics, and Permanent Education, University of Belgrade, Mike Petrovića Alasa, 11 000 Belgrade, Serbia

**Keywords:** thin Ni films, electrodeposition, biomass, rice husks, microstructure, composite hardness, sheet resistance

## Abstract

Lab-made biosilica (SiO_2_) nanoparticles were obtained from waste biomass (rice husks) and used as eco-friendly fillers in the production of nickel matrix composite films via the co-electrodeposition technique. The produced biosilica nanoparticles were characterized using XRD, FTIR, and FE-SEM/EDS. Amorphous nano-sized biosilica particles with a high SiO_2_ content were obtained. Various current regimes of electrodeposition, such as direct current (DC), pulsating current (PC), and reversing current (RC) regimes, were applied for the fabrication of Ni and Ni/SiO_2_ films from a sulfamate electrolyte. Ni films electrodeposited with or without 1.0 wt.% biosilica nanoparticles in the electrolyte were characterized using FE-SEM/EDS (morphology/elemental analyses, roundness), AFM (roughness), Vickers microindentation (microhardness), and sheet resistance. Due to the incorporation of SiO_2_ nanoparticles, the Ni/SiO_2_ films were coarser than those obtained from the pure sulfamate electrolyte. The addition of SiO_2_ to the sulfamate electrolyte also caused an increase in the roughness and electrical conductivity of the Ni films. The surface roughness values of the Ni/SiO_2_ films were approximately 44.0%, 48.8%, and 68.3% larger than those obtained for the pure Ni films produced using the DC, PC, and RC regimes, respectively. The microhardness of the Ni and Ni/SiO_2_ films was assessed using the Chen-Gao (C-G) composite hardness model, and it was shown that the obtained Ni/SiO_2_ films had a higher hardness than the pure Ni films. Depending on the applied electrodeposition regime, the hardness of the Ni films increased from 29.1% for the Ni/SiO_2_ films obtained using the PC regime to 95.5% for those obtained using the RC regime, reaching the maximal value of 6.880 GPa for the Ni/SiO_2_ films produced using the RC regime.

## 1. Introduction

Pure metallic materials in film or bulk forms, as well as metal matrix composite (MMC) coatings or films, such as Ni films reinforced with solid inorganic/organic inert particles [1], can be used in engineering applications where a high resistance to wear and tear and corrosion or a long device lifespan is required [2,3,4]. MMC films consist of soft and hard parts, where the soft part—or the matrix—is a metal or a metallic alloy, while the hard part or reinforcement consists of discrete inclusions dispersed throughout the softer part [5]. Generally, composite films can be classified into one of three groups based on the reinforcement microstructure: particle-reinforced MMC films, short-fiber filler or whisker filler MMC films, and continuous-fiber or sheet-reinforced MMC films [6].

The electrochemical deposition (ED) method is often used to produce pure and composite metallic films. The applied voltage or current density during the ED process; deposition time; cathode type; electrolyte composition, mixing conditions, and temperature influence the quality and structure of the electrodeposited metal and MMC films [7]. Electrolytes that are widely used for the deposition of pure nickel films are sulfamate [8], citrate and Watts’s [9], lactate [10], acetate [11], and the classic sulfate bath [12,13]. The application of a sulfamate electrolyte, compared to other electrolytes, results in very ductile Ni films with low internal stress and no sulfur (S) incorporation [8]. A sulfamate electrolyte consists of Ni-sulfamate salt, Ni-chloride salt, boric acid, and specific additives. The functions of the individual components in the sulfamate electrolyte are as follows: sulfamate salt controls the concentration of Ni ions, chloride salt helps with anode dissolution and enhances the electrical conductivity of the electrolyte, boric acid regulates the pH solution value and works as a weak buffer, and saccharine is the brightness additive [14].

Particles co-electrodeposited in a metallic matrix form MMC films, profoundly affecting the mechanical, chemical, and tribological characteristics of the coatings/films [15,16,17]. Numerous studies have utilized hard ceramic-based particles (Al_2_O_3_, ZrO_2_, and SiO_2_) [18], as well as carbides, such as SiC and WC, and lubricants in solid form (graphite) [19].

There are various methods for the production of MMC films, and all composite fabrication techniques can be classified into three basic categories: (1) liquid-phase processing (stir-casting, thermal spray, ultrasound-assisted stirring, semisolid stirring and squeeze casting, infiltration, and laser melt–particle injection), (2) solid-state processing (high-energy ball milling, diffusion bonding, cold spraying, powder metallurgy, and spark plasma sintering), and (3) reactive processing (an in situ reaction) [20]. The ED and co-electrodeposition (Co-ED) methods belong to the liquid-phase processing category for MMC fabrication [17].

While producing an MMC film using the Co-ED method, there are several problems that arise. Inadequate wettability between the inert ceramic particles and metal matrix, as well as very weak bonding between them, is the main problem. Other problems include unforeseen and unfavorable interface responses, a low particle concentration in the matrix, uneven and poor dispersion with unpredictable agglomeration, especially with surface-active nanoparticles (both in the electrolyte and in the film), and a low hydration force in the electrolyte [21,22]. Guglielmi theory [23] and modified Guglielmi particle transmission theory have been used to describe the kinetic model of the co-deposition of inert silica particles with a Ni matrix [24].

Recent trends in chemical and material engineering have focused on the utilization of eco-friendly chemicals as reinforcements in MMC films as part of a circular economy [25]. The conversion of biomasses into suitable nanoparticles, such as SiO_2_-, SiC-, and C-based materials, is preferred due to their environmentally friendly components. Given the low cost of raw materials and simple synthesis techniques, the utilization of agricultural waste to produce biosilica particles is becoming increasingly significant. Silica particles can be obtained from a variety of sources, including rice husks, wheat straw, sugar cane waste, corn cob ash, coconut husks, and grass. Approximately 96% of the silica found in the aforementioned sources comes from rice husks, providing a rationale for choosing rice husks to create silica particles [26]. Ekwenna et al. employed rice straw (RS) to extract amorphous silica using microbial digestion procedures [27]. Paredes et al. used a thermochemical technique to extract silica from agro-industrial waste biomass rice husks (RHs) [28]. Anuar et al. recovered SiO_2_ from coconut husks and investigated its microstructural characteristics [29]. Ramasamy et al. used a chemical technique to manufacture SiO_2_ from wheat straw [30]. Castano et al. created SiO_2_-based solids using the sol–gel technique and lemon bio-waste [31].

There are challenges associated with co-depositing SiO_2_ particles in a Ni matrix in baths with pH values higher than 3, which prevents the negatively charged SiO_2_ particles from being attracted to the cathode [32]. It is possible to hydrate the SiO_2_ oxygen surface layer to create silanol groups, which may interact with H^+^ or OH^-^ ions in the suspension [32]. This may potentially modify the surface charges of the particles. SiO_2_ nanoparticles and nickel have been successfully co-deposited from nickel sulfate-based baths [33] and acetate-based baths [34], as reported by several authors. According to Vidrich et al. [35], amorphous SiO_2_ nanoparticles can be incorporated onto the cathode and exhibit a positive zeta potential in sulfate baths with pH values between 2 and 6. MMC films of Ni/SiO_2_ were produced by Kasturibai et al. [34] using a nickel acetate bath, showing that the addition of SiO_2_ nanoparticles enhances the film’s microhardness and corrosion resistance. On the contrary, the use of surfactants, such as saccharine in the sulfamate electrolyte, aims to modify the particle surface charge. This is one method to increase the volume fraction of SiO_2_ nanoparticles in the MMC and achieve a homogenous distribution of the particles in the nickel matrix composite film.

All previous researchers utilized commercially available micrometric SiO_2_ particles [36,37,38]. In this study, SiO_2_ particles made in the lab using waste biomass. This low-cost technique for preparing biosilica particles used rice husks (RHs), resulting in the production of high-purity SiO_2_ nanoparticles. Obtaining high-purity biosilica (SiO_2_) nanoparticles was just the first goal of this investigation. In the next step, the synthesized biosilica nanoparticles were used as reinforcements for the production of nickel matrix composite (NiMC) films using various electrodeposition techniques. The formation of these films was the second goal of this investigation. ED/Co-ED methods were employed to produce pure Ni and NiMC films using different current regimes: direct current (DC), pulsating current (PC), and reversing current (RC) regimes. Hereafter, NiMC films are referred to as Ni/SiO_2_ films. For the production of Ni and Ni/SiO_2_ films, a sulfamate electrolyte with the addition of saccharine was used. The influence of biosilica reinforcements and current regimes on the Ni and Ni/SiO_2_ films was investigated with respect to their structural (FE-SEM), elemental (EDS/mapping), topographical (AFM), mechanical (Vickers hardness), and electrical (four-point probe method) properties. The true hardness of the Ni and Ni/SiO_2_ films was determined using the Chen-Gao composite hardness model (C-G CHM). Both the pure Ni and Ni/SiO_2_ films with biosilica nanoparticles synthesized from RHs were electrodeposited onto Si(100) substrates.

## 2. Materials and Methods

### 2.1. Materials and Methods for the Synthesis of SiO_2_ Nanoparticles from RH Biomass

For the preparation of nanosilica, rice husks were used, purchased from the rice producer Levidiagro in Kočani, North Macedonia. Sulfuric acid from Heicalium, produced by Zorka Šabac, Serbia, was used.

To remove contaminants, the rice husks were first washed with water and dried. They were then heated at 80 °C for three hours and treated with 10% sulfuric acid. The shell was subsequently torched, dried, and washed once more. The final step in synthesizing SiO_2_ nanoparticles from RH biomass involved a 4 h heat treatment in an oxidizing environment at 800 °C [39,40].

### 2.2. Materials and Methods for the Synthesis of Ni Films

For the preparation of pure Ni films, a lab-made sulfamate electrolyte was used. The p.a. reagents were purchased from Merck KGaA, Darmstadt, Germany, and doubly distilled water (Millipore, Burlington, MA, USA, 18 MΩ·cm) was used for the electrolyte preparation. The composition of the sulfamate electrolyte was as follows: 300 g/L Ni(NH_2_SO_3_)_2_·4H_2_O, 30 g/L NiCl_2_·6H_2_O, and 30 g/L H_3_BO_3_ [41]. A wetting surfactant, o-Benzoic sulfimide (i.e., saccharine), was added at 1 g/L. For mixing the sulfamate electrolyte without/with nanoparticles, a magnetic stirrer set to 100 rpm was used. The conditions for all current regimes are provided in Table 1. The surface area of the Si(100) cathode used for the ED/Co-ED processes was 2 cm^2^ (the dimensions were 1.0 × 2.0 cm), while the anode was a Ni sheet with 99.9% purity, situated close to the wall of the 100 mL Pyrex glass. The surface area of the Si(100) cathode for deposition was 2 cm^2^, while the anode was a Ni sheet with 99.9% purity, situated close to the wall of the 100 mL Pyrex glass.

The electrodeposition time was set to 300 s in all experiments. The film thicknesses were determined using a mechanical comparator, and the obtained values are provided in Table 1. The sulfamate electrolyte was heated to 50 ± 0.5 °C using hot plates with feedback. The pH value of the electrolyte was adjusted to 4.2 using a few drops of a 1 M solution of NaOH. To suppress the hydroxide precipitate at the bottom of the glass, a few drops of a 0.5 M acid solution (HCl) were gradually added. This combination enabled an optimum conductivity, metal ion concentration, and pH balance throughout the electrochemical processes.

The preparation of the Si(100) cathode for the ED and Co-ED processes was as follows: Si(100) cathodes were made from wafers (p-type; 4-inch; (100) orientation; 400 μm thick). The wafers were cleaned in a standard Piranha solution [42] and dried with N_2_ flow. Conducting layers on the Si(100) cathodes were obtained using spattering layers of 100 Å Cr, serving as an adhesion layer, and 1000 Å Au, serving as a nucleation layer. These sublayers provided adhesion and acted as a barrier to impurity diffusion.

### 2.3. Materials and Methods for the Synthesis of Ni/SiO_2_ Films

For the synthesis of Ni/SiO_2_ films, the biosilica SiO_2_ nanoparticles synthesized from rice husks were added at a concentration of 1.0 wt.% to the Ni sulfamate electrolyte. To ensure that the nanoparticles were homogeneously distributed throughout the sulfamate electrolyte, ultrasonic stirring was applied for 30 min in 3 cycles prior to the co-electrodeposition process. After homogenization of the electrolyte, magnetic stirring at 100 rpm was used during the co-electrodeposition process.

**Figure 1:**
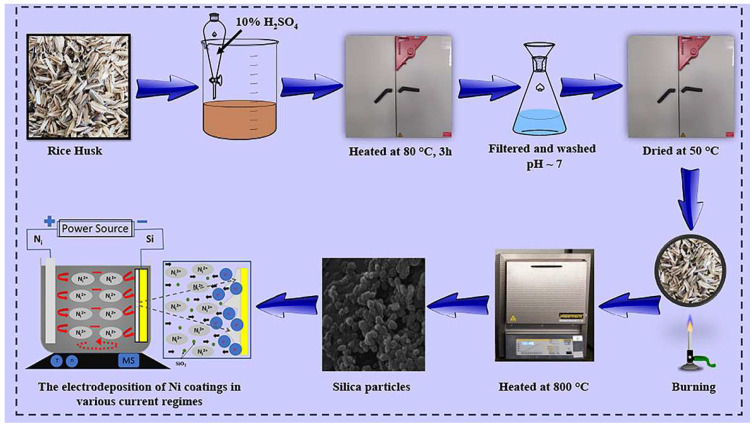
Experimental steps for the synthesis of biosilica nanoparticles and their co-deposition into a Ni matrix using the Co-ED process.

A scheme of the experimental work, including all of the synthesis steps, is shown in Figure 1.

### 2.4. Characterization Methods for SiO_2_ Nanoparticles

The microstructure of the particles was investigated using a 20 kV emission scanning electron microscope (FE-SEM Mira3 Tescan, Oxford, UK). The sample was sputtered with a thin layer of Au prior to imaging. EDS analysis was conducted using an INCAx-act LN2-free Analytical Silicon Drift Detector (Oxford Instruments, Oxford, UK), with the PentaFET^®^ Precision and Aztec 4.3 software package (Oxford Instruments, Oxford, UK), connected to the TESCAN Mira3 XMU. This FE-SEM was also utilized to investigate the structural characteristics of the pure Ni and Ni/SiO_2_ films.

To investigate the phase structure and crystallinity of the synthesized silica nanoparticles, X-ray diffraction (XRD) analysis was performed using an X-ray diffractometer (APD2000) in Bragg–Brentano geometry. The XRD investigation utilized Cu Kα radiation (λ = 1.5418 Å) with a 2θ angle range of 10–80°.

FTIR spectra of the SiO_2_ filler were acquired using a Nicolet 6700 spectrometer (Thermo Fisher Scientific, Waltham, MA, USA) in attenuated total reflectance (ATR) mode. The spectra were collected using ATR-corrected co-additions of 64 scans with a spectral resolution of 4 cm^−1^. The OMNIC 9 Paradigm software (Thermo Fisher Scientific, Waltham, MA, USA) captured spectra from 4000 to 400 cm^−1^.

### 2.5. Characterization Methods for Si(100) Cathode, Pure Ni, and Ni/SiO_2_ Films

The morphology of Ni electrodeposited with and without biosilica nanoparticles was examined using FE-SEM (the same type used for characterizing the biosilica nanoparticles).

A mechanical comparator with an electronic reader (model: Iskra, type: NP37; Iskra Avtomatica, Ljubljana, Slovenia) was used to determine the thickness of the films.

AFM characterization of the electrodeposited films was performed using the NTEGRA AFM device manufactured by NT-MTD (Moscow, Russia). The software used for the AFM measurements was NovaS, Image Analysis 2.2.0 (NT-MDT). AFM measurements were conducted using the semi-contact regime with a cantilever resonant frequency of 150 kHz. The cantilever was an NSG01, made from a single crystal silicon (n-type, 0.01–0.25 Ω·cm), doped with antimony, and coated with a reflective layer of Au. The set point during the AFM measurements was 60% of the free oscillation amplitude. All measurements were conducted with 256 points per line and 256 lines in total. The root mean square (*R*_q_ and *S*_q_) roughnesses of the films were determined using the free software Gwyddion 2.61 (Open-Source software, Czech Metrology Institute, Jihlava, Czechia) [43].

Vickers microhardness characterization of the obtained pure Ni and Ni/SiO_2_ films was performed using the Vickers microhardness tester “Leitz Kleinert Prufer DURIMET I” (Leitz, Oberkochen, Germany). The applied loads (P) were 5, 10, 15, 20, 25, 40, 50, 65, 90, 100, 150, 200, 250, and 300 gf (0.049–2.942 N), with a duration time (t) of 25 s. The composite hardness (H_c_) of the pure Ni and Ni/SiO_2_ films was calculated according to the standards ASTM E384 and ISO 6507 [44]. The Chen-Gao (C-G) composite hardness model (CHM) [45,46,47,48,49,50,51,52,53] was used to estimate the real hardness (H) of the pure Ni and Ni/SiO_2_ films. The real hardness of a film represents the hardness of the film on its own, with the influence of the hardness of the substrate eliminated. A Si(100) substrate coated with thin Cr/Au layers was utilized as the cathode in this investigation. The hardness of the substrate was calculated using the PSR (proportional specimen resistance) model [54] and was 6.49 GPa [55].

The four-point probe approach was used in a room setting to measure the sheet resistance of both the pure Ni and the Ni/SiO_2_ composite films. A Gwinstek GPS-3030D DC power supply (TME Group, Transfer Multisort Elektronik, Łódź, Poland) and a Keysight 34461A digital multimeter (Keysight Headquarters, Santa Rosa, CA) were utilized for this purpose [52]. The current intensity (*I*) was kept at a constant value of 45.3 mA. The probes were positioned on the film’s top surface. The average value of three separate measurements was taken and used to determine the final value of the film sheet resistance. The layer electrical conductivity was calculated from the measured resistance film thicknesses.

## 3. Results and Discussion

### 3.1. Characterization of SiO_2_ Nanoparticles

#### 3.1.1. Morphology of the SiO_2_ Nanoparticles

**Figure 2:**
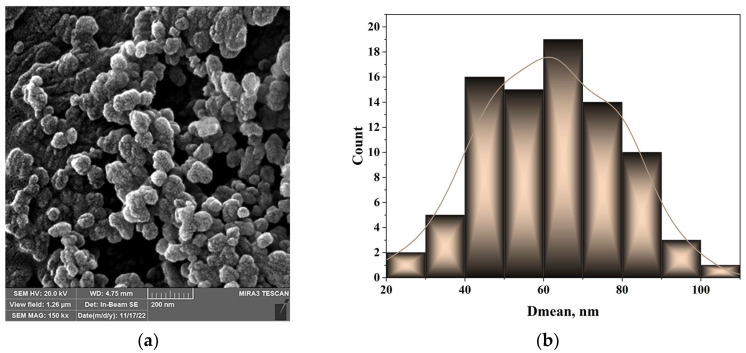
Morphology of the biosilica nanoparticles: (**a**) FE-SEM micrograph; (**b**) diameter distribution.

FE-SEM microphotography was used to analyze the acquired particles’ morphology, as displayed in Figure 2a, while the particle size distribution is shown in Figure 2b.

FE-SEM pictures were used to estimate the diameter distribution, which were then processed using the image analysis software Image Pro Plus 6.0 (Media Cybernetics, Rockville, MD, USA [56]. Based on the obtained histogram, the majority of the particles had diameters ranging from 50 to 100 nm.

#### 3.1.2. Elemental Analysis of the SiO_2_ Particles

**Figure 3:**
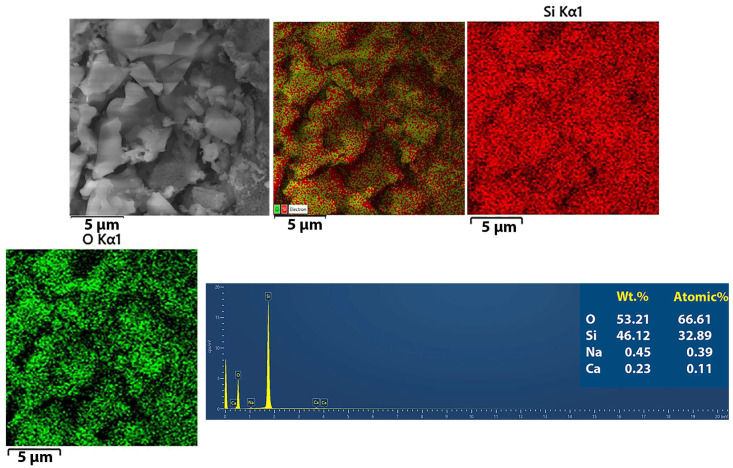
Silica particle elemental mapping, along with an EDS spectrum.

The FE-SEM images were also used to assess the elemental composition of the produced silica particles via EDS analysis (Figure 3).

Mapping analysis of the SiO_2_ nanoparticles confirmed a uniform distribution of Si and O atoms in the nanoparticles. The high purity of the synthesized biosilica was confirmed via EDS analysis since only traces of Na and Ca were detected in the nanoparticles.

#### 3.1.3. XRD Analysis of the SiO_2_ Particles

**Figure 4:**
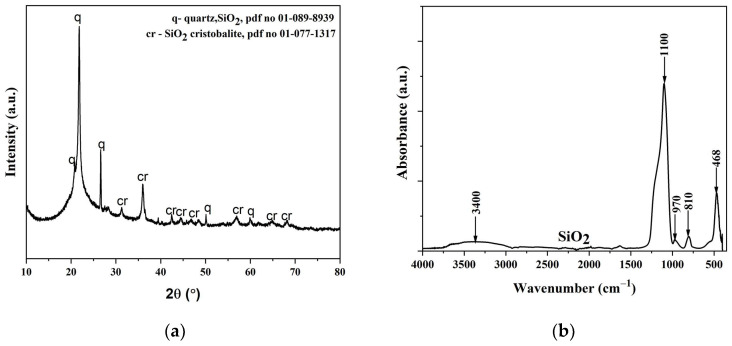
The synthesized silica particles characterization: (**a**) XRD spectrum; (**b**) FTIR spectrum.

An XRD diffractogram of the biosilica nanoparticles made from rice husks is shown in Figure 4a. The SiO_2_ particle FTIR spectrum is shown in Figure 4b.

The crystalline structure and chemical composition of the SiO_2_ under investigation were ascertained using XRD patterns. The diffractogram shows the spectrum of a solid material with crystal structures of quartz and cristobalite [57]. There is a dominant band at 22°, which is precisely the characteristic of SiO_2_ [57].

The OH group of molecular H_2_O exhibits stretching and in-plane bending vibrations, which result in absorption bands visible in the FTIR spectra at approximately 3400 cm^–1^ [58]. The spectra clearly show silica particle characteristic peaks at 970, 810, and 468 cm^−1^. The Si–O–Si elongation’s symmetric and antisymmetric vibrations can be attributed to absorption maxima of 1100 and 810 cm^−1^, respectively. The absorption peak at 468 cm^−1^ is associated with the vibration bending of the Si–O–Si bond [59].

### 3.2. Characterization of Ni and Ni/SiO_2_ Films

#### 3.2.1. The Basic Facts for Understanding the Current Regimes

The pulse reverse current (RC) regime is a common method involving periodically changing electrodeposition regimes, such as pulsating current (PC) and reversing current (RC) regimes [60]. The PC regime is defined by the average current density, *j*_av_, amplitude of the cathodic current density, *j_A_*, deposition time, *t_c_*, and pause duration, *t_p_*, as presented in Equation (1).

(1) $$j_{\mathrm{av}}=\frac{j_{A}\cdot t_{c}}{t_{c}+t_{p}}$$

The frequency of pulsating in this regime, ν_PC_, is defined in Equation (2).

(2) $$v_{\mathrm{PC}}=\frac{1}{t_{c}+t_{p}}$$

This regime provides optimum results in the millisecond range, in the (10–100) Hz range, in which electrodeposition occurs at the average current density [60,61].

The RC regime includes the anodic current density, *j_a_*, and the anodic time, *t_a_* (instead of a pause duration), and is represented by Equation (3).

(3) $$j_{\mathrm{av}}=\frac{j_{c}\cdot t_{c}-j_{a}\cdot t_{a}}{t_{c}+t_{a}}$$

where *j_c_* is the cathodic current density and *t_c_* is the cathodic time.

Analogous to the PC regime, the frequency, ν_RC_, in the RC regime is defined as follows:

(4) $$v_{\mathrm{RC}}=\frac{1}{t_{c}+t_{a}}$$

In the RC regime, the electrodeposition process also occurs at the average current density in the millisecond range. There is an unlimited number of combinations of parameters in the PC and the RC regimes by which the same average current density can be attained.

#### 3.2.2. Morphology of the Ni and Ni/SiO_2_ Films

**Figure 5:**
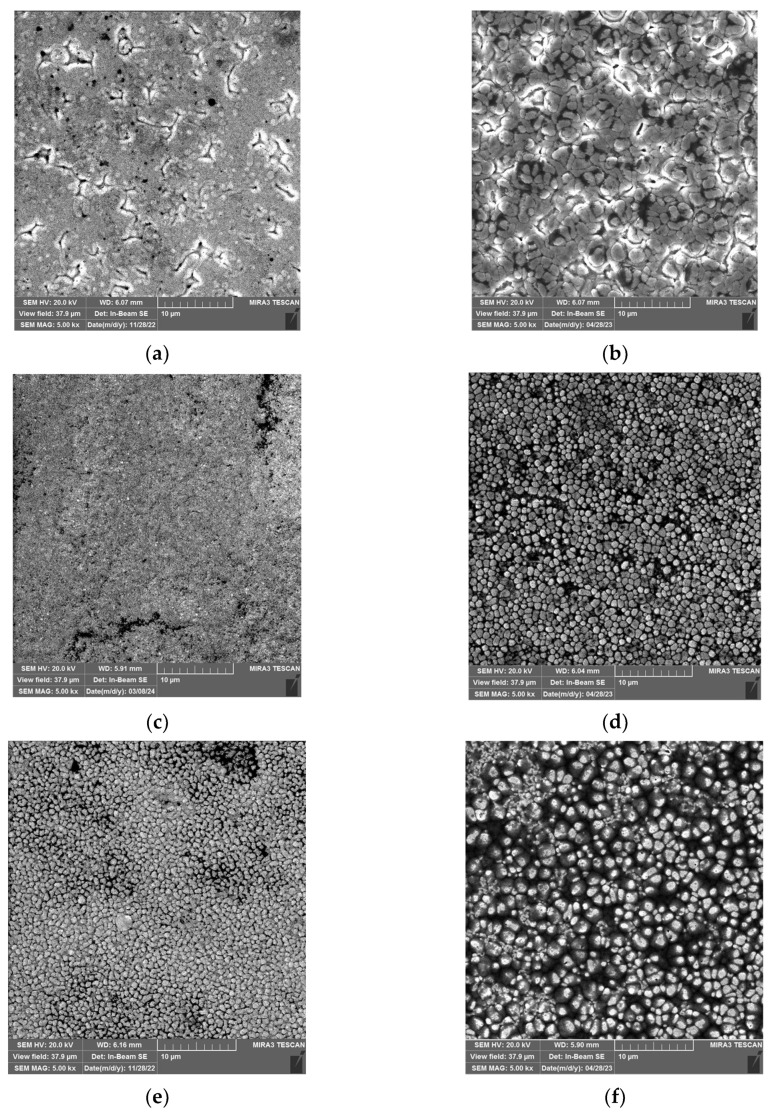
Surface morphologies of the electrodeposited pure Ni and Ni/SiO_2_ films prepared on the Si(100) cathode, with and without 1.00 wt.% biosilica nanoparticles, under different current regimes: (**a**) Ni-DC, (**b**) Ni/SiO_2_-DC, (**c**) Ni-PC, (**d**) Ni/SiO_2_-PC, (**e**) Ni-RC, and (**f**) Ni/SiO_2_-RC. The magnification was ×5000. The deposition time was 300 s. Electrodeposition processes were performed with a current density of 50 mA cm^−2^ in the DC regime and at the same average current density in the PC and RC regimes.

Figure 5 presents the surface morphology of the pure (silica-free) Ni films (Figure 5a,c,e) and the Ni/SiO_2_ films (Figure 5b,d,f) electrodeposited under varying current regimes: DC (Figure 5a,b), PC (Figure 5c,d), and RC (Figure 5e,f).

Upon comparing the Ni films obtained with and without biosilica nanoparticles under various regimes, it is clear that both the electrodeposition regimes and the addition of reinforcements to the sulfamate electrolyte had an influence on the microstructure of the Ni films. All Ni films are porous, likely due to hydrogen co-deposition as a parallel reaction. Grain boundaries are not noticeable in the pure Ni films obtained using the DC and PC regimes. The incorporation of biosilica nanoparticles resulted in Ni/SiO_2_ films with a more non-uniform morphology, i.e., coarser, compared to those without nanoparticles (Figure 5a,c vs. Figure 5b,d). This increased non-uniformity caused the formation of more pronounced grain boundaries. Furthermore, the spherical shape of the grains is evident in the films produced in the PC regime.

Very fine-grained Ni films were obtained using the RC regime, both with and without the addition of nanoparticles (Figure 5e,f). At first glance, the pure Ni films appear to have a more compact structure compared to the films without nanoparticles, which can be attributed to the incorporation of the nanoparticles into the film. A common characteristic of both types of film is the presence of approximately spherical grains.

**Figure 6:**
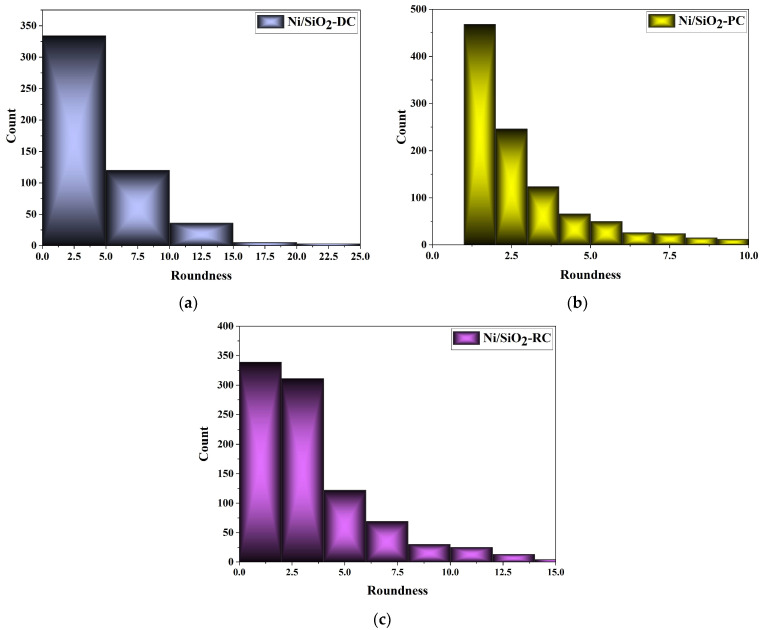
Roundness parameter obtained for the (**a**) Ni/SiO_2_-DC, (**b**) Ni/SiO_2_-PC, and (**c**) Ni/SiO_2_-RC regimes.

The appearance of approximately spherical grains in all of the Ni/SiO_2_ films necessitates additional analysis to examine the roundness of these films. Many investigations [62,63,64,65] have observed the dependence of grain size [65] in electrodeposited Ni films on variations in electrolyte type and composition, the presence of surfactants in electrolytes [63], electrolyte temperature [62], variations in current density in the DC regime [64], parameters in PRC regimes [66], and incorporated reinforcements in the metallic matrix [1,14,18,19,32,33,34,38]. However, there is a lack of data on the dependencies of grain roundness in metal films. Figure 6 shows the grain roundness distribution of Ni/SiO_2_ films electrodeposited from a sulfamate electrolyte with 1.00 wt.% biosilica using the DC (Figure 6a), PC (Figure 6b), and RC (Figure 6c) regimes.

The roundness parameter value of 1 corresponds to ideally spherical grains [15]. The maximum irregularity in grain shapes was observed for the Ni/SiO_2_ films synthesized in the DC regime, with roundness parameters in the 0–25 range (Figure 5b and Figure 6a). The highest sphericity of the grains was observed for the Ni/SiO_2_ composite films produced using the PC regime (Figure 5d and Figure 6b). The roundness parameter values for Ni/SiO_2_ deposited in the RC regime were in the 0–15 range (Figure 5f and Figure 6c). This indicates that, in addition to spherical grains, there were also grains with sharp edges. The estimated roundness parameters are in accordance with the observed morphology of the Ni/SiO_2_ films.

#### 3.2.3. Mapping of the Ni and Ni/SiO_2_ Films—EDS

**Figure 7:**
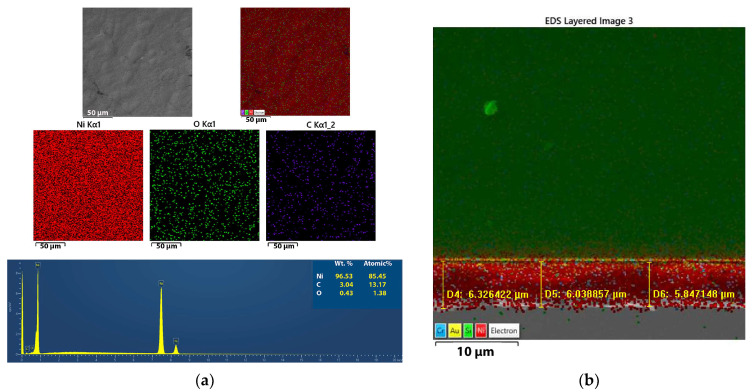
(**a**) Elemental mapping/EDS spectrum and (**b**) cross-sectional analyses of the electrodeposited silica-free Ni films prepared on a Si(100) substrate in the PC regime.

**Figure 8:**
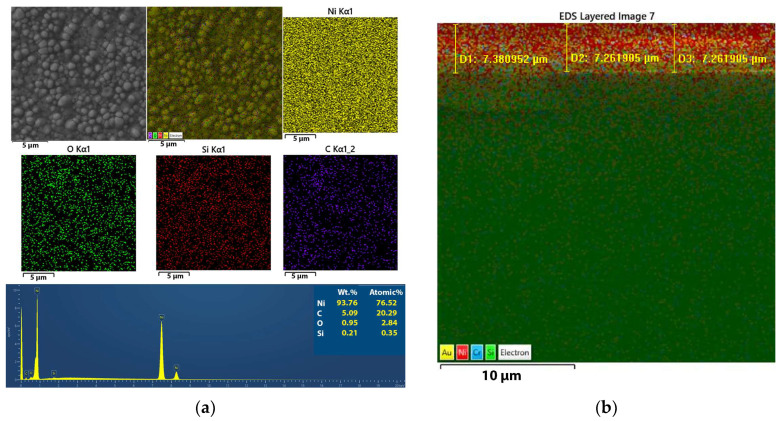
(**a**) Elemental mapping/EDS spectrum and (**b**) cross-sectional analyses of the electrodeposited Ni/SiO_2_ films prepared on a Si(100) substrate in the PC regime.

Elemental mapping analysis of the Ni films formed with and without biosilica nanoparticles was carried out in order to prove that changes in the surface morphology can be attributed to the incorporation of biosilica nanoparticles in these films. This analysis was also used to assess the composition and purity of the films, as illustrated in Figure 7a for the pure Ni films and Figure 8a for the Ni/SiO_2_ composite films, both of which were obtained using the PC regime. In Figure 7b and Figure 8b, the appropriate cross-sections with mapping of the samples are also provided.

Based on the spectral analysis in Figure 7a, as expected, a high percentage of Ni (96.53%) was obtained in this film. The presence of carbon and oxygen is common in Ni films, probably originating from saccharine.

The elemental mapping analysis revealed that the Ni/SiO_2_ films consisted of Ni, C, O, and Si (Figure 8a). This visual representation clearly shows the arrangement of various elements within the composite films. Elemental mapping analysis confirmed the successful incorporation of biosilica nanoparticles into the films.

#### 3.2.4. Topography of the Ni and Ni/SiO_2_ Films

**Figure 9:**
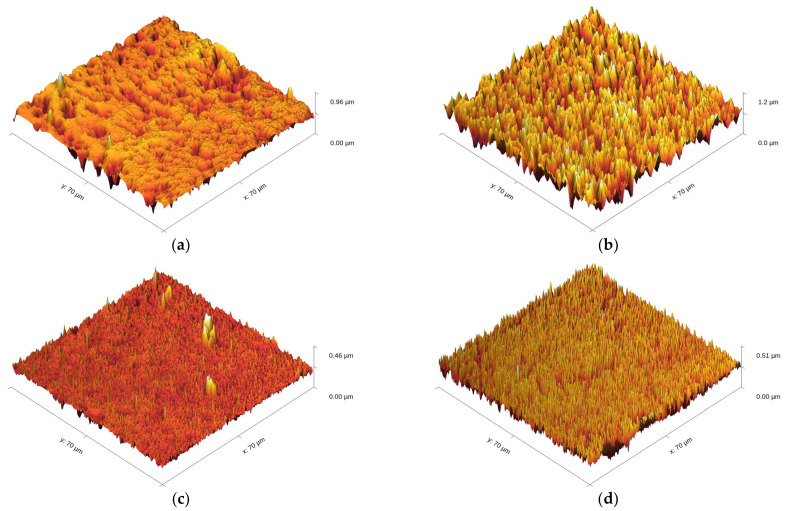

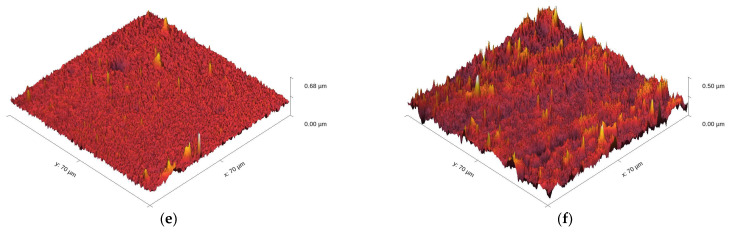
Three dimensional (3D) AFM images of the electrodeposited pure Ni and Ni/SiO_2_ films, prepared on Si(100) cathodes with or without 1.00 wt.% biosilica nanoparticles, under different current regimes: (**a**) Ni-DC, (**b**) Ni/SiO_2_-DC, (**c**) Ni-PC, (**d**) Ni/SiO_2_-PC, (**e**) Ni-RC, and (**f**) Ni/SiO_2_-RC. The scan size was 70 × 70 μm^2^. The deposition time was 300 s. Electrodeposition processes were performed at a current density of 50 mA cm^−2^ in the DC regime, and at the same average current densities in the PC and RC regimes.

A further comparison of the Ni films obtained with and without biosilica nanoparticles under various electrodeposition regimes was carried out using the AFM technique. Figure 9 represents the 3D (three-dimensional) pictures of the pure Ni films (Figure 9a,c,e) and Ni/SiO_2_ films (Figure 9b,d,e) with varying current regimes: DC (Figure 9a,b), PC (Figure 9c,d), and RC (Figure 9e,f).

The root mean square roughness parameter (*R*_q_) values, obtained using AFM software Gwyddion 2.61 (Open Source, Czech Metrology Institute, Jihlava, Czechia), for the Ni films produced with or without biosilica nanoparticles are provided in Table 2.

The roughness of the Ni films increased with the incorporation of biosilica nanoparticles into the sulfamate electrolyte, and this trend was consistent across all three regimes (Table 2). Regarding the surface roughness (*S*_q_) values, these increased by 44.0% for the Ni films obtained using the DC regime, 48.8% for the Ni films obtained using the PC regime, and 68.3% for those obtained using the RC regime. These increases in film roughness are due to the incorporation of nanoparticles in the deposit and their agglomeration during the co-deposition process, which resulted in higher roughness for the Ni films with incorporated nanoparticles. Among the Ni/SiO_2_ composite films, the highest roughness was obtained in the DC regime (105.4 nm), which decreased with the application of PRC regimes, thereby confirming the benefits of using PRC regimes in ED processes.

#### 3.2.5. Microhardness Properties of the Ni and Ni/SiO_2_ Films Using the Chen-Gao Composite Hardness Model

The Chen-Gao composite hardness model (C-G CHM) [46,47,48,49,50,51,52,53,54] was utilized to assess the true hardness of the deposited films, independent of the substrate hardness. The dependency of the measured composite hardness (*H*_c_), including the contribution of the substrate hardness (in this case, Si(100)) on the indentation depth, *h*, is described in Equation (5) according to the C-G CHM.

(5) $$H_{c}=A+B\cdot \frac{1}{h}+C\cdot \frac{1}{h^{n+1}}$$

where *A*, *B,* and *C* are the fitting parameters; *h* can be calculated as 1/7 of the diagonal size; *n* is the constant of the C-G CHM, named the “power index” (*n* = 1.8 or *n* = 1.2, depending on the system type).

The real film hardness is expressed according to Equation (6) [46].

(6) $$H=A\pm \sqrt[{n}]{\frac{{\left[n\cdot \left|B\right|/\left(n+1\right)\right]}^{n+1}}{n\cdot \left|C\right|}}$$

**Figure 10:**
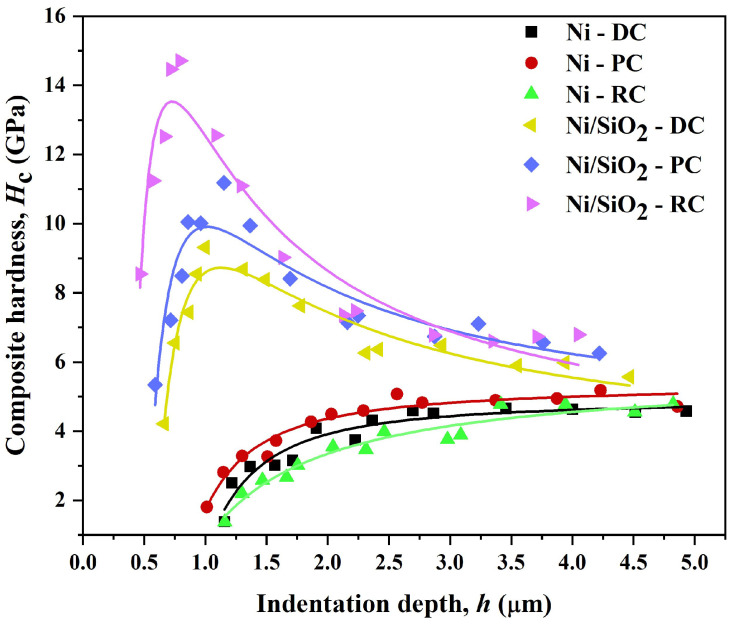
Composite hardness characteristics of pure Ni and Ni/SiO_2_ films deposited on Si(100) substrates under varying current deposition regimes (DC, PC, and RC). The fitting curves resulted from the application of the Chen -Gao composite hardness model.

Figure 10 displays the fitting of experimental microhardness data for the Ni and Ni/SiO_2_ films using the C-G CHM. The dependences of *H*_c_ on *h* for the pure Ni films show a clear increase in composite hardness with indentation depth, indicating that these films belong to the “soft film on hard substrate” composite system type [15,41]. However, a very interesting behavior was observed for the Ni/SiO_2_ films. For these films, the composite hardness initially increased, reaching a maximum at a specific indentation depth, and then decreased exponentially, approaching the Si(100) hardness value of 6.49 GPa. It is clear that the maximal *H*_c_ value represents the transition from a zone where the film’s hardness predominantly influences the measured composite hardness to a zone where the substrate’s hardness becomes the major contributing factor. Hence, the maximum value of composite hardness signifies the transition from the dominant influence of the film hardness to that of the substrate hardness.

The intrinsic or real hardness (*H*) values for all films, calculated according to Equation (6), together with the other parameters, are provided in Table 3. This confirms that the C-G CHM was successfully applied for the quantitative estimation of the real hardness of both pure Ni and Ni/SiO_2_ films deposited electrochemically on the Si(100) substrate.

Table 3 shows that the pure Ni films obtained in the DC, PC, and RC regimes exhibit lower real hardness values than their Ni/SiO_2_ counterparts. The increases in the hardness of the Ni/SiO_2_ films relative to the pure Ni films were as follows: 37.2% for Ni films obtained using the DC regime, 29.1% for Ni films obtained using the PC regime, and 95.5% for those obtained using the RC regime. The observed Vickers hardness value for Ni/SiO_2_ composites of 620 HV (6.08 GPa) [67] coincides well with the values found in this investigation. For the Ni/SiO_2_ films, the hardness increased in the order Ni/SiO_2_-DC → Ni/SiO_2_-PC → Ni/SiO_2_-RC, indicating the benefits of using PRC regimes, i.e., demonstrating a close correlation between grain size (morphology) and film hardness. However, an unexpected behavior was observed for the pure Ni films, where the film obtained using the RC regime was thinner than those obtained using the DC and PC regimes. This is likely due to the reduced thickness of this Ni film caused by dissolution during the anodic component of the RC regime (Equation (3)).

The increase in the microhardness of Ni films with incorporated biosilica nanoparticles can be explained as follows: the dispersion of SiO_2_ nanoparticles in the film hinders the dislocation movement, thereby strengthening the composite film and preventing grain boundary slip effects [68]. SiO_2_ nanoparticles, as a second phase in sulfamate electrolytes, more efficiently lower the nucleation energy of nickel crystals, leading to an increased number of nucleation sites, thus helping to slow down the growth rate of nickel and decrease grain size [67]. The increase in the hardness of the Ni/SiO_2_ films compared to the pure Ni films can also be correlated with the increased roughness of the films as follows: greater roughness means a higher number of grain boundaries in the films, which act as mini resistors and reduce the indentation depth during microindentation. The smaller the indentation depth, the greater the hardness observed in the film.

#### 3.2.6. Electrical Properties of the Ni and Ni/SiO_2_ Films—Sheet Resistance

To examine the influence of electrodeposition regimes and the incorporation SiO_2_ particles on the electrical properties of Ni films, the sheet resistance *R* (Ω/□) of Ni films obtained with and without nanoparticles was measured using the four-point probe method. The sheet resistance values of the Ni and Ni/SiO_2_ films, as well as the calculated electrical conductivity *σ* (S/cm), are provided in Table 4.

Generally, factors affecting the measured values of sheet resistance *R* (Ω/□) of electrochemically produced metallic films include the film thickness, surface morphology, substrate type (conductivity), incorporation of impurities from the electrolyte, type of post-treatment process (e.g., annealing), roughness, and the incorporation of ceramic particles [69,70,71,72]. While the mechanical characteristics of Ni and Ni/SiO_2_ films are highly dependent on the ED and Co-ED process parameters, the electrical properties tend to remain relatively constant despite changes in the plating conditions.

Controlling the desired thickness of thin layers through electrodeposition regimes is complex, especially for RC regimes (where thinner layers are typically obtained due to the presence of the anodic components) and when there is the incorporation of inert particles in the films (thicker layers are then obtained). Based on the data in Table 4, it can be concluded that the sheet resistance of the Ni/SiO_2_ films obtained in all three regimes was lower than that obtained for the pure Ni films. This decrease can be explained as follows: Luo et al. [73] showed that the electrical resistivity of ED Ni films is around three times higher than that of bulk Ni or very thick Ni films. This is because surface scattering contributes more to the film resistivity as the film thickness decreases, and further increases in film resistivity occur due to electron scattering at grain boundaries if the metal layer contains polycrystalline grains [74]. The Ni islands start to expand and merge as the film grows, reducing the distance between them and creating a network of linked islands. As a result, the probability of electrons hopping from one island to other increases quickly, and this lowers film resistance. This observation supports the comparison of Ni films with and without nanoparticles under identical electrodeposition regimes. All Ni films reinforced with biosilica showed a lower sheet resistance than pure Ni films produced using the same regime.

### 3.3. Mechanism of Formation of Nickel Matrix Composite (NiMC) Films and Comparison with Existing Data

**Figure 11:**
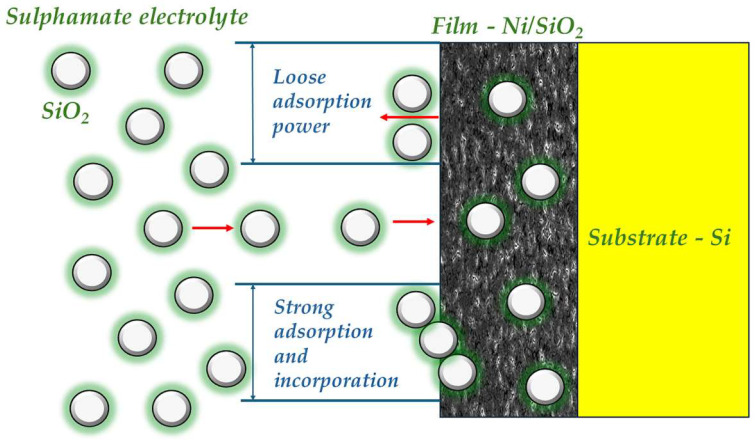
A schematic of the co-deposition process of biosilica nanoparticles from a sulfamate electrolyte in a Ni film according to the Guglielmi model.

The “trapping mechanism”, which predicts the incorporation of inert particles suspended in an electrolyte [23,24] into metal matrix composite coatings, can be used to explain the formation of Ni matrix composite films with biosilica nanoparticles. According to the adsorption mechanism proposed by Guglielmi [23,24], there are two consecutive steps involved in the complete incorporation of SiO_2_ nanoparticles into a metal film. In the first step, during the co-deposition process, SiO_2_ particles are loosely adsorbed onto the cathode surface as they travel around it, leading to their incorporation into the film and increasing the particle content with longer electrodeposition times. The Langmuir adsorption isotherm suggests that there is a high degree of surface coverage due to van der Waals forces, which weakly adsorb particles onto the cathode surface [75]. In the second step, because of the applied electric field, the particles are pushed by Coulomb forces onto the surface and into the growing metal matrix [75]. The concept of these two stages—strong and weak adsorption—on the electrode surface is illustrated in Figure 11.

Previous investigations have explored the formation of Ni films/coatings using electrodeposition techniques with commercially available SiO_2_ particles of various dimensions. Nickel matrix composite (NiMC) films have been electrodeposited from both deep eutectic solvents [22] and aqueous electrolytes [24,32,34,35], using different electrodeposition regimes and parameters, such as electrolyte type and composition, the presence of additives in the electrolyte, the current density applied, and the type of cathode. Among other things, an increase in the hardness of Ni films has been observed in some of these investigations [32,34]. Since an increase in hardness has been reported for Ni/SO_2_ films produced from electrolytes different from those used in this study—such as sulfate baths (Watts bath) with varying concentrations of polyethyleneimine (PEI) as a cationic surfactant [32] and acetate baths [34]—it is evident that the observed increase in hardness of Ni films is primarily due to the incorporation of SiO_2_ particles, rather than other electrodeposition parameters. The results obtained in this study using biosilica are in accordance with those found using commercially available SiO_2_.

## 4. Conclusions

Biosilica nanoparticles were produced from rice husks and used as reinforcements (1.0 wt.%) in the production of nickel matrix composite (Ni/SiO_2_) films using various electrodeposition regimes (direct constant current (DC), pulsating current (PC), and reversing current (RC) regimes). Based on a comparison of the morphological, mechanical, and electrical features between the Ni/SiO_2_ and pure Ni films, it can be concluded that SiO_2_ nanoparticles were successfully incorporated into the Ni matrix and that they fulfill the conditions to be used as reinforcements in Ni ED/Co-ED processes.

Although the morphology of the Ni films was primarily determined using the electrodeposition regime, the incorporation of SiO_2_ nanoparticles also strongly contributed to the surface features of the films. As a result of the incorporation of nanoparticles, Ni films with clear boundaries among grains were formed in the DC and PC regimes, while the Ni films obtained in all three regimes were rougher than the pure Ni films. Depending on the applied electrodeposition regime, the surface roughness of the Ni/SiO_2_ films was greater than that obtained for the pure Ni films, between 44.0% (for the films obtained using the DC regime) and 68.3% (for the films obtained using the RC regime). Simultaneously, the microhardness of the Ni/SiO_2_ films was greater, while their sheet resistance was lower than that obtained for the pure Ni films. The hardness of the Ni films with incorporated biosilica nanoparticles was increased compared to that of the pure Ni films, with an enhancement ranging from 29.1% for the Ni/SiO_2_ films produced in the PC regime to 95.5% for the Ni/SiO_2_ films produced in the RC regime. The maximum hardness of 6.880 GPa was obtained for the Ni/SiO_2_ films electrodeposited using the RC regime, which was 41.5% and 23.7% greater than the hardness values of the Ni/SiO_2_ films produced in the DC and PC regimes, respectively.

## Figures and Tables

**Table 1:** The synthesized samples and electrochemical deposition parameters. DC—constant current regime; PC—pulsating current regime; RC—reversing current regime; *j*—current density in the DC regime; *j_A_*—amplitude of the current density; *j_c_*—cathodic current density; *j_a_*—anodic current density; *δ*—film thickness; *t_c_*—deposition time; *t_p_*—pause duration; *t_a_*—anodic time; *ν*_PC_—frequency of pulsating in the PC regime; *ν*_RC_—frequency of pulsating in the RC regime; *j*_av_—average current density.

Film Type/Regime	*j*/mA·cm^−2^	*j_A_* or *j_c_/j_a_*/mA·cm^−2^	*δ*/μm	*t_c_*/ms	*t_p_*/ms (in PC), and *t_a_* (in RC)	*ν*_PC_ or *ν*_RC_/Hz	*j*_av_/mA·cm^−2^
Ni/DC	50	/	6.5 ± 0.3	/	/	/	/
Ni/SiO_2_/DC	50	/	6.8 ± 0.4	/	/	/	/
Ni/PC	/	100	6.3 ± 0.2	10	10	50	50
Ni/SiO_2_/PC	/	100	7.2 ± 0.6	10	10	50	50
Ni/RC	/	100/100	5.3 ± 0.2	15	5	50	50
Ni/SiO_2_/RC	/	100/100	7.5 ± 0.7	15	5	50	50

**Table 2:** The root mean square roughness parameters (*R*_q_ and *S*_q_) obtained using Gwydion software 2.61 from 70 × 70 µm^2^ scans of the Ni films, both with and without silica nanoparticles, under various current regimes.

Film Type/Regime	Ni-DC	Ni/SiO_2_-DC	Ni-PC	Ni/SiO_2_-PC	Ni-RC	Ni/SiO_2_-RC
*R*_q_/nm	78.36 ± 3.13	105.4 ± 5.13	37.87 ± 2.03	49.02 ± 2.95	32.26 ± 2.26	46.66 ± 3.21
*S*_q_/nm	73.67 ± 3.13	106.1 ± 5.13	32.45 ± 1.13	48.28 ± 2.95	27.87 ± 1.26	46.90 ± 3.01

**Table 3:** Calculated intrinsic hardness values of the Ni and Ni/SiO_2_ films, *H* (in GPa), deposited on the Si(100) cathodes, together with the fitting parameters (*A*, *B*, *C*) and standard error (*SE*) values.

Film Type/Regime	Ni-DC	Ni/SiO_2_-DC	Ni-PC	Ni/SiO_2_-PC	Ni-RC	Ni/SiO_2_-RC
*A* ± SE	3.225 ± 0.4	4.971 ± 0.4	3.956 ± 0.6	5.405 ± 0.3	3.029 ± 0.6	5.741 ± 0.3
*B* ± SE	9.663 ± 0.8	−1.139 ± 1.3	9.396 ± 1.2	−1.439 ± 0.8	11.913 ± 1.0	−4.724 ± 1.1
*C* ± SE	−4.293 ± 0.4	−3.37 ± 1.3	−3.444 ± 0.4	−2.251 ± 0.7	−2.409 ± 0.3	−0.209 ± 1.2
*H*	3.542	4.861	4.308	5.562	3.519	6.880

**Table 4:** Sheet resistance values (*R* in Ω/□) for pure Ni and Ni/SiO_2_ films produced on Si(100) substrates, measured using the four-point probe method. *U*—voltage in mV measured between the inner probe tips in three locations (*U*_1_, *U*_2_, and *U*_3_); *U*_av_—average voltage; *σ*—electrical conductivity (in S/cm).

Film Type/Regime	Ni-DC	Ni/SiO_2_-DC	Ni-PC	Ni/SiO_2_-PC	Ni-RC	Ni/SiO_2_-RC
*U*_1_/mV	394	330	383	156	360	212
*U*_2_/mV	429	320	410	170	355	180
*U*_3_/mV	360	296	460	180	360	180
*U*_av_/(mV)	394.3	315.3	417.7	168.7	358.3	190.7
*R*/(Ω/□)	39.43	31.53	41.77	16.87	35.83	19.07
*σ*/(S/cm)	39.02	46.64	38.00	82.52	52.66	69.92

## Data Availability

The data presented in this study are available on request from the corresponding author or co-authors. The data are not publicly available.
